# Clinical observation of probiotics combined with mesalazine and Yiyi Baitouweng Decoction retention enema in treating mild-to-moderate ulcerative colitis

**DOI:** 10.1515/med-2024-1126

**Published:** 2025-02-25

**Authors:** Yanlong Li, Baoyu Li, Yuqin Gou, Xudong Tian, Lijun Chang, Chaoxu Qu

**Affiliations:** Diagnosis and Treatment Center for Spleen and Stomach Diseases, Gansu Provincial Hospital of Traditional Chinese Medicine, Lanzhou 730030, Gansu, China; Endocrinology Department of Spleen and Stomach Diseases, Xigu District Traditional Chinese Medicine Hospital, Lanzhou 730060, Gansu, China; Chronic Non communicable Disease Control Institute, Gansu Provincial Center for Disease Control and Prevention, Lanzhou 730000, Gansu, China; College of Integrated Chinese and Western Medicine, Gansu University of Traditional Chinese Medicine, Lanzhou 730000, Gansu, China

**Keywords:** ulcerative colitis, probiotic, mesalazine, Yiyi Baitouweng Decoction retention enema, clinical observation

## Abstract

**Objective:**

The purpose of this article was to unravel the clinical efficacy of probiotics combined with mesalazine and Yiyi Baitouweng Decoction retention enema in the treatment of mild-to-moderate ulcerative colitis (UC).

**Methods:**

Eighty-six mild-to-moderate UC patients were selected as study subjects and randomized into the control group (treated with mesalazine enteric-coated tablets [Salofalk]) and the observation group (treated with mesalazine enteric-coated tablets, Bifidobacterium Tetravaccine Tablets, and Yiyi Baitouweng Decoction retention enema). The clinical efficacy, colonoscopy score, serum levels of inflammatory factors, and the incidence of adverse reactions were compared.

**Results:**

The clinical efficacy of patients in the observation group was better in contrast to the control group. After 8 weeks of treatment, the colonoscopy score, and levels of TNF-α, IFN-γ, CRP, and ESR were lower, while IL-10 levels were higher in patients of both groups than those before treatment; lower colonoscopy score and levels of TNF-α, IFN-γ, CRP, and ESR and higher IL-10 levels were observed in the observation group versus the control group.

**Conclusion:**

Probiotics combined with mesalazine and Yiyi Baitouweng Decoction retention enema have remarkable clinical effects in treating mild-to-moderate UC.

## Graphical abstract

**Figure:**
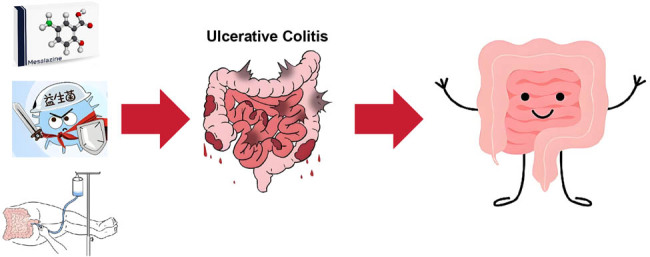

## 1 Introduction

Ulcerative colitis (UC), known as a chronic inflammatory bowel disease (IBD), is able to involve any aspect of the colon beginning with the mucosal inflammation of the rectum and proximally extending in a continuous manner [1]. Most UC patients show a mild moderate course, usually most active during diagnosis and then in different remission or mild activity [2]. The etiology of UC involves interactions among the environment, gut microbiome, and immune system, as well as genetic susceptibility to disease [3]. UC cannot be diagnosed definitively via any single diagnostic study. Instead, this disease is based on an overall interpretation of the laboratory tests, and clinical manifestations, together with histological, endoscopic, and radiological findings [4]. Treatment options for UC are usually based on the pattern of disease involvement and the extent of its clinical activity [5]. More and more people have recognized that tailoring treatment strategies to the demands of patients can improve patient compliance and thus reduce relapse rates [6].

The primary treatment for mild-moderate UC is the 5-aminosalicylic acid (5-ASA) class drugs, consisting of mesalamine and sulfasalazine, along with diazo-bonded 5-ASA [7]. In both oral and topical formulations, mesalazine has shown efficacy in inducing active colitis and maintaining remission irrespective of the extent of inflammation [8]. Some previous trials have demonstrated its superiority for inducing remission and maintenance therapy in the treatment of UC when compared with placebo and rectal steroids [9,10]. However, physicians should monitor emerging inflammatory reactions (e.g., crampy abdominal pain, myalgias, arthralgias, bloody diarrhea, and/or erythematous skin rashes) for each patient with mesalazine during each follow-up visit [11]. Interaction of the host inflammatory response and the gut microbiota has revealed that alternative therapies (e.g., probiotics) play complementary roles in the treatment and prevention of disease flares. A variety of probiotics together with their formulations have been investigated to induce and maintain remission in UC [12]. A long-term treatment approach of anti-inflammatory drugs combined with probiotics is feasible and can be used as an alternative to corticosteroids in treating mild-to-moderate UC [13]. Currently, complementary and alternative agents are increasingly utilized for treating IBD because of their potential efficacy, and they account for approximately 21% of IBD patients now [14]. Among them, Baitouweng Decoction is a classic herbal medicine formula widely applied clinically in China for the treatment of intestinal-related diseases [15]. It has been utilized in China for the treatment of UC for hundreds of years, achieving remarkable clinical effects. Research data indicate that Baitouweng Decoction can ameliorate clinical symptoms and histopathological damage in UC mice, while also reducing the release of proinflammatory cytokines, including interleukin (IL)-6, IL-1β, and tumor necrosis factor (TNF)-α [16]. Xie et al. similarly demonstrate that Baitouweng Decoction exhibits good efficacy and safety in treating UC, improving symptoms of colitis damage and inhibiting inflammatory responses [17]. This study focused on Yiyi Baitouweng Decoction, the prescription composition of which comprises Baitouweng, Huangbai, Huanglian, Qinpi, Yiyiren, Diyu, Wubeizi, Baijiangcao, and Cheqianzi. Among these, Yiyiren, the dried mature seed of Coicis Semen (a plant of the Gramineae family), possesses properties such as invigorating the spleen to eliminate dampness, alleviating arthralgia and pain, clearing heat, and promoting the discharge of pus. The purpose of this article was to unravel the clinical efficacy of probiotics combined with mesalazine and Yiyi Baitouweng Decoction retention enema in the treatment of mild-to-moderate UC.

## 2 Materials and methods

### 2.1 General data

Eighty-six patients who visited Xigu District Traditional Chinese Medicine Hospital from January 2023 to January 2024 with a diagnosis of mild or moderate UC were selected for the study. Inclusion criteria were as follows: those who met the diagnostic criteria for UC in the Consensus Opinion on the Diagnosis and Treatment of Inflammatory Bowel Disease (2018-Beijing); those whose diagnosis was confirmed by pathohistology and enteroscopy; those whose degree of the disease was mild or moderate, and whose disease stage was active; those whose age was ≥18 years old; and those whose clinical data were complete. Exclusion criteria were as follows: those with severe UC; those who were allergic to the drugs used in this research; those who had a combination of other intestinal diseases; those who had a combination of malignant tumors; those who had a combination of severe cognitive dysfunction; those who had used glucocorticosteroids, immunosuppressant agents, and biologics in the last month prior to the enrollment; and those who were pregnant or lactating. The patients were randomized into the control group and the observation group, with 43 cases in each group. The general information of the patients in the two groups was comparable, and the difference was not statistically significant (Table 1, *P* > 0.05).

**Table 1:** Comparison of general information of the patients in the two groups (*x̄* ± SD) [*n* (%)]

Group	Gender		Mean age (years)	Mean course of disease (years)	Disease severity	
	Male	Female			Mild	Moderate
Control group (*n* = 43)	25 (58.14)	18 (41.86)	40.47 ± 7.35	3.28 ± 0.92	29 (67.44)	14 (32.56)
Observation group (*n* = 43)	20 (46.51)	23 (53.49)	40.09 ± 6.68	3.54 ± 1.06	27 (62.79)	16 (37.21)
*P* value	0.388		0.714	0.088	0.821	

### 2.2 Treatment methods

All patients were given conventional treatments such as anti-inflammatory, analgesic, and nutritional support to maintain water and electrolyte balance.

Patients in the control group were treated with mesalazine enteric-coated tablets (Salofalk; CMS China Medical system, 0.5 g × 40 tablets), 1.0 g/dose, taken orally 1 h before meals, 4 times/day for 8 weeks.

The patients in the observation group were treated with Bifidobacterium Tetravaccine Tablets (Siliankang; Hangzhou Yuanda Biopharmaceutical Co., Ltd; specification: 0.5 g × 24 tablets) + Yiyi Baitouweng Decoction retention enema on the basis of the control group. For the Bifidobacterium Tetravaccine Tablets, patients were instructed to take three tablets/time, with warm water orally 30 min after meals, and 3 times/day; for Yiyi Baitouweng Decoction retention enema, the prescription consisted of 30 g of Baitouweng, 20 g of Huangbai, 20 g of Huanglian, 20 g of Qinpi, 20 g of Yiyiren, 20 g of Diyu, 15 g of Wubeizi, 10 g of Baijiangcao, and 15 g of Cheqianzi. Each dose of the decoction was boiled and concentrated to approximately 100 mL (prepared uniformly by our hospital’s decoction room). Before use, the decoction was heated to 38–40°C, and patients were instructed to empty their bowels before the enema. Then, patients were instructed to take the knee–chest position, choose the appropriate enema tube, insert it into the anus at 10–15 cm, and slowly inject the decoction into the body; during the injection process, patients were instructed to elevate the buttocks. Then, patients were advised to lie on their left and right sides for 30 min each to allow the decoction to fully distribute over the ulcerated area in the intestine and retain the decoction in the body for 1 h, once before bedtime a day, for 8 weeks. Both groups were followed up for 3 months.

### 2.3 Testing indicators and methods

Clinical efficacy evaluation was as follows: after 8 weeks of treatment, the clinical efficacy of the two groups of patients was evaluated based on the results of the colonoscopy review and the relevant determination criteria of UC in the Consensus Opinion on Diagnosis and Treatment of Inflammatory Bowel Diseases (2018 – Beijing). Apparent effectiveness was as follows: after treatment, the patient’s clinical symptoms such as abdominal pain, diarrhea, and gastrointestinal bleeding disappeared, and the colonoscopy results showed that the intestinal mucosa returned to normal without congestion or erosion and did not recur within 3 months; effectiveness was as follows: after treatment, the patient’s clinical symptoms significantly improved, colonoscopy results showed the presence of mild congestion, edema symptoms of the intestinal mucosa, reduced intestinal mucosal inflammatory reaction, as well as the presence of a few erosive ulcers or pseudo-polyps; ineffectiveness was as follows: no significant change or deterioration of clinical symptoms and colonoscopy findings of patients after treatment. Total effective rate = (number of apparent effective + number of effective)/total number of cases × 100%. In this study, colonoscopy was performed by the same endoscopist and this endoscopist was unaware of the grouping of patients.

Colonoscopy scoring was as follows: colonoscopy was performed before treatment and after 8 weeks of treatment, using the Rachmilewitz endoscopic scoring system. The scoring criteria were as follows: granularity: none: 0 points, yes: 2 points; vascular distribution: normal vascular distribution: 0 points, vague and disturbed vascular distribution: 1 point, complete disappearance of blood vessels: 2 points; mucosal damage: no damage: 0 point, mild damage: 2 points, severe damage: 4 points; brittle and fragile mucous membranes: no change: 0 points, a mild increase: 2 points, a significant increase: 4 points. The total score was 0–12, with higher scores indicating a more severe condition of the patient.

Measurement of serum inflammatory factor levels was as follows: all patients were taken 5 mL of peripheral elbow venous blood on an empty stomach in the early morning before and after treatment, centrifuged at 3,000 rpm for 10 min for the separation of the supernatant. The serum levels of TNF-α, interferon (IFN)-γ, and IL-10 were assessed by enzyme-linked immunosorbent assay, the serum levels of C-reactive protein (CRP) were tested by immunoturbidimetric assay, and the levels of erythrocyte sedimentation rate (ESR) were detected by ESR meter, detected with product matching test tubes and anticoagulants. The reagent kits used in the experiments were procured from Sangong (Shanghai, China) following the instructions of the kits.

Adverse reactions were as follows: The occurrence of adverse reactions such as nausea and vomiting, headache, dizziness and fatigue, abdominal distension, and liver function abnormality was observed during the treatment of the two groups of patients, and the incidence rate of adverse reactions = the number of cases of adverse reactions/the total number of cases × 100%.

### 2.4 Data statistics

All data were statistically analyzed with SPSS 21.0 (IBM SPSS Statistics, Chicago, IL, USA) and GraphPad Prism 9.0 statistical software (GraphPad Software, Inc., La Jolla, CA, USA). Measurement data were tested to conform to normal distribution and satisfied homogeneity of variance, which were expressed as mean ± standard deviation (SD). For comparisons within groups, the paired-samples *t*-test was adopted, while for comparisons between groups, the independent-samples *t*-test was employed. The categorical data were expressed as rate (%) and analyzed by Fisher’s exact test. *P* < 0.05 was considered to be a statistically significant difference.


**Informed consent:** The patients and their families voluntarily participated in the research and signed a written informed consent form.
**Ethical approval:** The study got approval from the Ethics Committee of Xigu District Traditional Chinese Medicine Hospital (approval number: 2022091).

## 3 Results

### 3.1 Clinical outcomes between the two groups of patients after treatment

After treatment, a higher total effective rate of patients was observed in the observation group versus the control group (*P* < 0.05; Table 2).

**Table 2:** Comparison of the clinical efficacy of patients between the two groups [*n* (%)]

Group	Apparent effective	Effective	Ineffective	Total effective rate (%)
Control group (*n* = 43)	10 (23.26)	23 (53.48)	10 (23.26)	76.74
Observation group (*n* = 43)	22 (51.56)	19 (44.19)	2 (4.65)	95.35
*P* value				0.0261

### 3.2 Comparison of colonoscopy scores before and after treatment

Before treatment, no statistical significance was observed in the colonoscopy scores of patients in the two groups (*P* > 0.05). At the end of the 8-week treatment, the colonoscopy scores of patients in the two groups were notably reduced compared with the pre-treatment period, and the scores of the patients in the observation group were lower in contrast to those in the control group (*P* < 0.05; Table 3).

**Table 3:** Comparison of colonoscopy scores before and after treatment between the two groups (point, *x̄* ± SD)

Group	Before treatment	After treatment	*P* value
Control group (*n* = 43)	8.18 ± 2.29	4.52 ± 1.32	<0.001
Observation group (*n* = 43)	8.09 ± 2.25	2.14 ± 0.88	<0.001
*P* value	0.926	<0.001	

### 3.3 Comparison of inflammatory factor levels before and after treatment

No significant difference was noted in the comparison of serum TNF-α, IFN-γ, IL-10, CRP, and ESR levels of patients between the control and the observation groups before treatment (*P* > 0.05). After treatment, serum TNF-α, IFN-γ, CRP, and ESR levels were lower and IL-10 levels were higher than those before treatment in both groups, and lower TNF-α, IFN-γ, CRP, and ESR levels and higher IL-10 levels were found in the observation group in contrast to the control group (*P* < 0.05; Table 4).

**Table 4:** Comparison of serum inflammatory factor levels before and after treatment (*x̄* ± SD)

Group	Time	TNF-α (ng/L)	IFN-γ (ng/mL)	IL-10 (ng/mL)	CRP (mg/L)	ESR (mm/h)
Control group (*n* = 43)	Before treatment	58.24 ± 13.23	13.25 ± 3.29	20.86 ± 6.24	63.58 ± 12.82	42.75 ± 12.36
	After treatment	44.18 ± 10.14*	9.64 ± 1.80*	30.41 ± 8.63*	27.31 ± 5.36*	21.69 ± 4.98*
Observation group (*n* = 43)	Before treatment	56.77 ± 12.82	12.98 ± 3.17	22.61 ± 6.92	61.66 ± 11.52	41.60 ± 11.14
	After treatment	36.36 ± 9.56*^#^	7.13 ± 1.44*^#^	37.29 ± 9.16*^#^	19.96 ± 4.27*^#^	13.33 ± 3.46*^#^

Note: *P < 0.05 vs before treatment within the same group; ^#^ *P* < 0.05 vs control group.

### 3.4 Comparison of the occurrence of adverse reactions between the two groups

During the treatment period, a total of seven cases of adverse reactions such as nausea and vomiting, headache, dizziness and fatigue, and abdominal distension occurred in the control group patients, and a total of four cases of adverse reactions such as nausea and vomiting, headache, dizziness and fatigue, and abdominal distension occurred in the observation group. No statistical significance was noted in the incidence of adverse reactions between the two groups of patients (*P* > 0.05; Table 5).

**Table 5:** Comparison of adverse reactions in the two groups [*n* (%)]

Group	Nausea and vomiting	Headache	Dizziness and fatigue	Abdominal distension	Total incidence rate (%)
Control group (*n* = 43)	3 (6.98)	2 (4.65)	1 (2.33)	1 (2.33)	7 (16.28)
Observation group (*n* = 43)	2 (4.65)	1 (2.33)	1 (2.33)	0	4 (9.30)
*P* value					0.520

## 4 Discussion

The emergence of novel therapeutics and better recognition of the biological pathways of the disease have led to improved management of UC patients [18]. Side effects of systemic steroids, non-adherence to medications, and practice variability among physicians represent barriers to limiting the optimal therapeutic management of patients with mild-to-moderate UC [19]. As therapeutic targets become more ambitious, encompassing symptomatic, endoscopic, and histological remission, clinicians must optimize the use of existing treatments and promptly identify patients who may benefit from transitioning to more intensive therapies [20,21]. Herein, we aimed to unravel the clinical efficacy of probiotics combined with mesalazine and Yiyi Baitouweng Decoction retention enema in the treatment of mild-to-moderate UC.

As the first-line therapy for mild-to-moderate UC patients, mesalazine’s action and delivery mechanisms are pivotal for the successful management of the disease [22]. With the development of the medical levels along with the deepening of research, UC has been disclosed to correlate with the imbalance of the intestinal flora. The supplement of probiotics to the therapeutic process has become a novel therapeutic concept and approach [23]. As previously reported, probiotics improve the immune system and intestinal mucosa barrier functions and enhance anti-inflammatory factor secretion, thereby restraining the harmful bacteria growth in the intestine [24]. Meanwhile, UC patients taking probiotics exhibit fewer severe symptoms and slow up the symptom severity of patients [25]. According to data reports, the combination of Bifidobacterium Tetravaccine and mesalazine for the treatment of UC in China has shown satisfactory efficacy, with superior safety compared to the use of mesalazine or Bifidobacterium Tetravaccine alone [26]. Baitouweng Decoction, a traditional Chinese medicine prescription, has a long history of use in treating UC. It has been demonstrated that Baitouweng Decoction can significantly improve inflammatory symptoms in mice with acute colitis, with its underlying mechanisms potentially related to multiple signaling pathways, including the regulation of intestinal microbiota and inflammatory signaling pathways [27]. Furthermore, Zhao et al. have confirmed that Baitouweng Decoction has a significant adjuvant therapeutic effect on UC patients, reducing tumor necrosis factor levels in these patients. They also reveal that the combination of traditional Chinese medicine and mesalazine is more effective in treating UC than mesalazine alone [28].

In this study, a control group treated with mesalazine enteric-coated tablets and an observation group receiving mesalazine enteric-coated tablets, Bifidobacterium Tetravaccine Tablets, and Yiyi Baitouweng Decoction retention enema were established. The results unearthed that compared to the control group, the observation group exhibited a higher total effective treatment rate, lower colonoscopy scores, after 8 weeks of treatment, lower levels of TNF-α, IFN-γ, CRP, and ESR, and higher IL-10 levels. This implied that the combination of probiotics, mesalazine, and Yiyi Baitouweng Decoction retention enema was more effective in treating mild-to-moderate UC than mesalazine alone.

To conclude, this research suggests that the combination therapy of probiotics, mesalazine, and Yiyi Baitouweng Decoction retention enema has a remarkable clinical effect in mild-to-moderate UC, which can effectively reduce the patient’s colonoscopy scores, lessen inflammatory levels, and has a small risk of adverse reactions. This article supplies an evidence-based regimen for the management of UC. Probiotics/mesalazine/Yiyi Baitouweng Decoction may be a novel treatment for UC. However, this study lacks a group without Bifidobacterium Tetravaccine Tablets to compare the effects of Yiyi Baitouweng Decoction versus mesalazine alone. Therefore, it is imperative to conduct high-quality, multifaceted, and long-term follow-up studies in the future.
